# From monocyte‐derived macrophages to resident macrophages—how metabolism leads their way in cancer

**DOI:** 10.1002/1878-0261.13618

**Published:** 2024-02-27

**Authors:** Ummi Ammarah, Andreia Pereira‐Nunes, Marcello Delfini, Massimiliano Mazzone

**Affiliations:** ^1^ Laboratory of Tumor Inflammation and Angiogenesis, Center for Cancer Biology VIB Leuven Belgium; ^2^ Laboratory of Tumor Inflammation and Angiogenesis, Department of Oncology, Center for Cancer Biology KU Leuven Belgium; ^3^ Department of Molecular Biotechnology and Health Sciences, Molecular Biotechnology Centre University of Torino Italy; ^4^ Life and Health Sciences Research Institute (ICVS), School of Medicine University of Minho Braga Portugal; ^5^ ICVS/3B's‐PT Government Associate Laboratory Braga/Guimarães Portugal

**Keywords:** cancer metabolism, immunometabolism, monocyte‐derived macrophages, tissue‐resident macrophages, tumor microenvironment, tumor‐associated macrophages

## Abstract

Macrophages are innate immune cells that play key roles during both homeostasis and disease. Depending on the microenvironmental cues sensed in different tissues, macrophages are known to acquire specific phenotypes and exhibit unique features that, ultimately, orchestrate tissue homeostasis, defense, and repair. Within the tumor microenvironment, macrophages are referred to as tumor‐associated macrophages (TAMs) and constitute a heterogeneous population. Like their tissue resident counterpart, TAMs are plastic and can switch function and phenotype according to the niche‐derived stimuli sensed. While changes in TAM phenotype are known to be accompanied by adaptive alterations in their cell metabolism, it is reported that metabolic reprogramming of macrophages can dictate their activation state and function. In line with these observations, recent research efforts have been focused on defining the metabolic traits of TAM subsets in different tumor malignancies and understanding their role in cancer progression and metastasis formation. This knowledge will pave the way to novel therapeutic strategies tailored to cancer subtype‐specific metabolic landscapes. This review outlines the metabolic characteristics of distinct TAM subsets and their implications in tumorigenesis across multiple cancer types.

## Graphical abstract

Tumor‐associated macrophages (TAMs) constitute up to 50% of the tumor mass, representing a heterogeneous population of tissue‐resident and monocyte‐derived macrophages. TAM phenotype not only involves alterations in cell metabolism but also metabolic reprogramming that can dictate their activation state and function. This review elucidates the diverse roles and metabolic traits of distinct TAM subsets in pancreatic, breast, lung and ovarian malignancies.

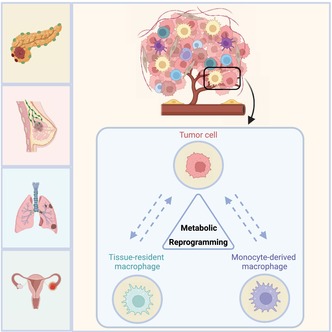

**Abbreviations**
2‐DG: 2‐deoxyglucose
AA: amino acid
AM: alveolar macrophage
AMP: adenosine monophosphate
APC: antigen‐presenting cell
ARG1: arginase‐1
BMDM: bone marrow‐derived macrophage
CCL18: CC chemokine ligand 18
CSF‐1: colony‐stimulating factor‐1
dC: deoxycytidine
DCN1: decorin 1
DM: ductal macrophage
EGF: epidermal growth factor
EMT: epithelial–mesenchymal transition
ETC: electron transport chain
FA: fatty acid
FABP: fatty acid‐binding protein
FBP1: fructose bisphosphatase 1
GARP: glycoprotein‐A repetitions predominant
GLUT1: glucose transporter 1
GPR132: G‐protein‐coupled receptor 132
HGF: hepatocyte growth factor
HIF1‐α: hypoxia‐inducible factor 1‐alpha
HUVEC: human umbilical vein endothelial cell
ICAM‐1: intercellular adhesion molecule‐1
IFN‐γ: interferon‐gamma
IL‐1: interleukin‐1
iNOS: inducible nitric oxide synthase
IntM: interstitial macrophage
IRG1: immune responsive gene 1
LCN‐2: lipocalin‐2
LUAD: lung adenocarcinoma
MAPK: mitogen‐activated protein kinase
MDM: monocyte‐derived macrophage
MMP‐9: matrix metalloproteinase‐9
mTORC1: mammalian target of rapamycin complex 1
NADPH: nicotinamide adenine dinucleotide phosphate
NAM: nerve‐associated macrophage
nCB: carbon black ultrafine
NF‐κB: nuclear factor kappa b
NO: nitric oxide
NSCLC: nonsmall‐cell lung cancer
OXPHOS: oxidative phosphorylation
PanIN: pancreatic intraepithelial neoplasia
PBMC: peripheral blood mononuclear cell
PDAC: pancreatic ductal adenocarcinoma
PDO: patient‐derived organoid
PEM: peritoneal macrophage
PGC‐1α: peroxisome proliferator‐activated receptor gamma coactivator‐1 alpha
PGE2: prostaglandin E2
PITPNM3: phosphatidylinositol transfer protein 3
PPARγ: peroxisome proliferator‐activated receptor gamma
PPP: pentose phosphate pathway
PUFA: polyunsaturated fatty acid
ROS: reactive oxygen species
RXR: retinoid X receptor
SM: stromal macrophage
STAT3: signal transducer and activator of transcription 3
TAM: tumor‐associated macrophage
TCA: tricarboxylic acid cycle
TDE: tumor‐derived exosome
TGF‐β: transforming growth factor‐beta
TME: tumor microenvironment
TNBC: triple‐negative breast cancer
TNF‐α: tumor necrosis factor‐alpha
TRM: tissue‐resident macrophage
VCAM‐1: vascular cell adhesion molecule‐1
VEGF: vascular endothelial growth factor

## 1 Introduction

**Fig. 1:**
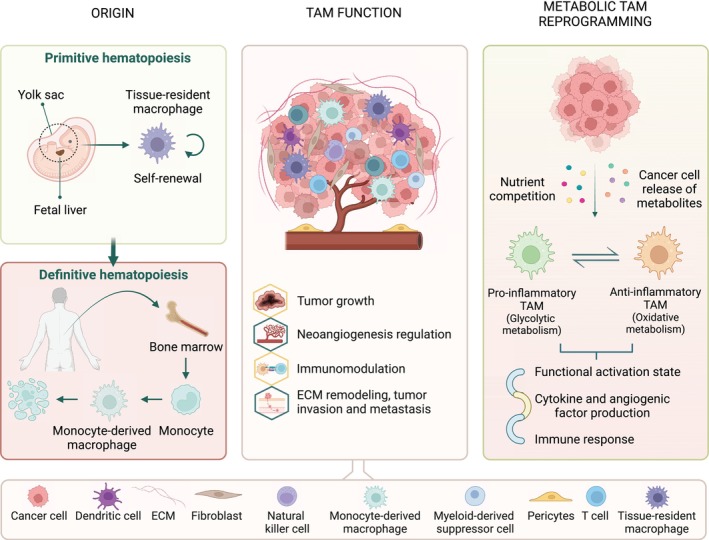
Macrophage origin, function, and metabolic reprogramming in cancer. Tissue‐resident macrophages develop in the embryonic yolk sac and fetal liver during waves of primitive hematopoiesis and self‐maintain throughout adulthood. Postnatally macrophage populations are continuously replenished by definitive hematopoietic progenitors raised in the bone marrow, which upon stimulation, differentiate into monocyte‐derived macrophages in peripheral tissues (left panel). Tissue‐resident and monocyte‐derived macrophages comprise a heterogenous and the most abundant population in several cancer types and contribute to tumor growth, angiogenesis, metastasis, and immunosuppression (middle panel). During tumor progression, macrophages are metabolically reprogrammed by cancer cells to support their proliferative demands and consequently, acquire specific functions and activation profiles (right panel). TAM, tumor‐associated macrophage.

Macrophages are effector cells of the innate immune system with evolutionarily conserved roles in tissue development and homeostasis [1]. Based on their origin, macrophages are broadly categorized into tissue‐resident macrophages (TRMs) and monocyte‐derived macrophages (MDMs). TRMs are seeded during early phases of development and self‐renew to persist throughout an individual's lifespan. They maintain tissue homeostasis and integrity by acting as immune sentinels during development and postnatally [1, 2, 3, 4, 5]. In turn, MDMs derive from circulating monocytes that exit the bloodstream and undergo differentiation into macrophages within various tissues (Fig. 1). These macrophages contribute to the maintenance of tissue‐specific macrophage pools and exert key functions during inflammation [6, 7]. In some organs, such as the lungs, liver, kidney, and brain, MDMs co‐exist with TRMs [8].

Macrophages exhibit remarkable phenotypic and functional diversity and are involved in the regulation of immune responses and inflammation. Following an infection or injury, Ly6C^+^ inflammatory monocytes are recruited from the blood, and differentiated into macrophages as they migrate towards the affected tissues [6]. In the early stages of healing, these recruited macrophages are often proinflammatory in nature and secrete a variety of inflammatory factors, such as tumor necrosis factor alpha (TNF‐α), interleukin 1 (IL‐1), nitric oxide (NO) and reactive oxygen species (ROS), to activate antimicrobial defense mechanisms [9]. However, these proinflammatory molecules can also trigger some tissue damage because of the toxic activity of ROS and NO. In these circumstances, macrophages undergo apoptosis or switch to an anti‐inflammatory phenotype in order to counteract the tissue‐damaging potential of prolonged inflammatory responses [10, 11]. However, in response to several chronic insults, macrophages can switch from being the regulators of homeostasis to being aides in the development of different diseases [10, 11]. Therefore, the regulatory mechanisms involved in macrophage repolarization play a major role in the progression and resolution of many diseases, including cancer [12, 13, 14, 15].

In the context of cancer, macrophages are usually referred to as tumor‐associated macrophages (TAMs). TAMs constitute a heterogenous population of tissue‐resident cells and a large proportion of recruited MDMs that, in a majority of tumors, constitute up to 50% of the tumor mass [16]. Increased macrophage infiltration into the tumor microenvironment (TME) is generally associated with a poor prognosis in most human cancers and promotes tumor progression, metastasis, and angiogenesis (Fig. 1) [17, 18, 19, 20].

At the tumor onset, macrophages are able to eliminate tumor cells through several mechanisms, such as antibody‐dependent cellular cytotoxicity, phagocytosis, induction of vascular damage and tumor necrosis, as well as activation of innate and adaptive immune responses. However, once the tumor is established, macrophages typically aid in the tumor progression by supporting cancer cell survival and proliferation, promoting angiogenesis, and suppressing immune cell‐mediated cytotoxicity (Fig. 1) [16, 21, 22]. These processes are essential for the transformation of tumor cells into a metastatic state [23]. Numerous studies have shown the involvement of both TRMs and MDMs in tumorigenesis however, it remains uncertain which of these exerts a more significant influence [24]. The lack of clear phenotypic markers that can differentiate TAMs based on their origin restricts functional investigations [25]. Recent studies have suggested that TRMs may exhibit distinct functions and phenotypes from their MDM counterparts in different types of cancer. TRMs are the first to be subject to the effects of tumor‐produced soluble factors, TME insults, and early inflammatory changes, which ultimately prompts them to facilitate the recruitment of MDMs and generation of TAMs [26]. Once tumors progress past the initial stage, the role of TRMs seems to be organ specific with some cancer models showing a decrease of these populations over time, whereas in others, they seem to favor cancer cell growth [27]. On the other hand, MDMs seem to change their transcriptional profile when encountering TME conditions and generally adopt a more protumoral, angiogenic, and immunosuppressive phenotype that supports tumor progression [26]. Moreover, both TRMs and MDMs seem to foster the formation of a premetastatic niche and facilitate cancer cell engraftment at the metastatic sites (Fig. 1) [16, 21, 22, 28].

Accumulating evidence over the last decade has shown that, not only is TAM activation coupled with metabolic alterations but the metabolic profile of TAMs can also dictate their function and phenotype. In this respect, metabolic reprogramming of TAMs has recently gained increased attention as it represents a novel approach to “re‐educate” their functions from protumoral to anti‐tumoral in a specific TME [27, 29, 30, 31, 32, 33]. Nonetheless, the appreciation of the dynamic nature of macrophage metabolism in cancer is relatively new and much remains to be studied.

In this review, we describe the metabolic features of tissue‐resident and monocyte‐derived macrophages in pancreatic, lung, breast and ovarian cancers as well as cell‐specific metabolic interventions to harness TAMs for tumor prevention and treatment. The rationale for selection of these particular cancer types was dictated by the available research literature on the metabolic attributes of TAM subsets within these specific malignancies.

## 2 Metabolic landscape of macrophages

Macrophage metabolism is very dynamic and known to be rewired in response to the nutritional demands of cancer cells and TME disturbances [34]. In fact, TAMs adopt distinct polarization signatures upon stimulation with different TME metabolic cues, which are accompanied by alterations in glucose, amino acid, lipid, and iron metabolism [35, 36, 37]. Such metabolic rewiring is functionally important for TAM survival as well as to determine their functional outcome as it can facilitate the production of cytokines and angiogenic factors and can reshape both intracellular signaling and intercellular communications (Fig. 1) [38, 39].

An increased glycolytic metabolism, fatty acid (FA) synthesis, pentose phosphate pathway (PPP) activation, and a truncated tricarboxylic acid (TCA) cycle are associated with an M1‐like macrophage activation state, characterized by antitumor and proinflammatory functions. The enhanced glycolytic metabolism generates the amount of ATP required for macrophage secretory and phagocytic functions and facilitates the forwarding of the carbon flux to oxidative PPP, which will, in turn, provide intermediate products necessary for amino acid and nucleotide synthesis [40]. Additionally, this pathway constitutes a major source of nicotinamide adenine dinucleotide phosphate (NADPH), which can be used to produce both ROS and NO, further exacerbating the tumoricidal potential of macrophages. Furthermore, the disrupted TCA cycle leads to the accumulation of TCA intermediates, such as citrate, itaconate, and succinate that sustain macrophage inflammatory responses by increasing the production of lipids, prostaglandins, NO, and ROS mediators [41, 42]. Finally, it has been shown that high levels of succinate stabilize hypoxia‐inducible factor 1 alpha (HIF1‐α) and induce the secretion of proinflammatory cytokines, such as IL‐1β [43].

Conversely, rewiring of macrophage metabolism towards oxidative phosphorylation (OXPHOS), FA oxidation and glutamine metabolism is associated with protumoral and anti‐inflammatory/M2‐like macrophages. Protumoral macrophages boost FA oxidation to generate acetyl‐CoA from long‐chain fatty acids, which is then oxidized through the TCA cycle and electron transport chain (ETC) to produce energy, or transferred into the cytoplasm to regulate cytosolic NADPH [44]. Glutamine metabolism contributes to the anaplerotic replenishment of the TCA cycle as it provides carbon and nitrogen for the synthesis of amino acids, proteins, nucleotides, and lipids [45]. Notably, it has been reported that glutaminolysis‐derived α‐ketoglutarate is involved in the destabilization of HIF1‐α, enhanced FA oxidation, and in the epigenetic reprogramming of TAMs [46]. In addition to these bioenergetic pathways, the catabolism of arginine, via arginase1 (ARG1), is favored over nitric oxide synthetase (iNOS) in protumoral macrophages, resulting in the production of tumor‐supporting factors, such as ornithine and polyamines [47, 48].

Considering the complexity and dynamism of the TME and the plasticity of TAMs, it is clear that the static vision of pro‐inflammatory/M1‐like and anti‐inflammatory/M2‐like macrophage classification and characterization may not fully describe the metabolic features and events that occur within a tumor. Therefore, niche‐specific conditions as well as the temporal and spatial structure of metabolic rewiring during immune responses should be considered when studying macrophage metabolism.

### 2.1 Pancreatic cancer

Macrophages residing in the pancreas exhibit distinct phenotypes and functions depending on their intrapancreatic location and micro‐environmental stimuli. Pancreatic macrophages can be broadly categorized into two main groups: those located within the islets of Langerhans and those situated in the interacinar stroma. Macrophages found in the islets of Langerhans are derived from definitive hematopoiesis, are present in the islets since birth, undergo local proliferation and exhibit a more proinflammatory phenotype [49, 50]. These macrophages can be further subdivided into two subsets based on the expression of CD11c. The CD11c^−^ macrophages are primarily situated in the peri‐islet region, while the CD11c^+^ macrophages are predominantly found within the islet structure [51]. On the other hand, macrophages in the interacinar stroma broadly exhibit an anti‐inflammatory phenotype and are categorized based on the expression of CD206 and CD301. The CD206^−^CD301^−^ macrophages are originated from definitive hematopoiesis, whereas the CD206^+^CD301^+^ macrophages derived from primitive hematopoiesis and are primarily located around the pancreatic ducts [49, 50]. Interestingly, the distinct features of pancreatic macrophage populations are maintained even after replacement with donor stem cells using genotoxic methods, confirming that pancreatic anatomy dictates the function of macrophages [50].

In pancreatic ductal adenocarcinoma (PDAC), macrophages compose the most abundant immune cell population and play a pivotal role in tumor development and progression [52]. At early stages of tumor progression, inflammatory macrophages are stimulated by KRAS‐mutated acinar cells to release proinflammatory cytokines, including TNF‐α and IL‐6. These cytokines trigger acinar to ductal metaplasia by initiating specific signaling pathways, such as nuclear factor kappa‐light‐chain‐enhancer of activated B cells (NF‐kB), mitogen‐activated protein kinase (MAPK) and signal transducer and activator of transcription 3 (STAT3) [53, 54, 55]. Consequently, proinflammatory macrophages are eventually reprogrammed toward an antitumoral phenotype. In line with this, it was reported that in late‐stage pancreatic intraepithelial neoplasias (PanINs) and invasive PDAC, anti‐inflammatory macrophages dominate the TME [54, 55, 56, 57, 58]. Of note, increased macrophage density has been associated with poor prognosis and survival in PDAC patients [52, 59, 60].

In PDAC tissues, both TRMs and MDMs were shown to contribute to the TAM pool; however, the former were indicated as more potent drivers of PDAC progression. An interesting study form Zhu et al. [61] identified monocyte‐derived infiltrating macrophages as tumor antigen‐presenting cells (APCs) and regulators of tumor immunosuppression and adaptive immunity, whereas TRMs were shown to exhibit unique profibrotic transcriptional profiles, which might fine‐tune PDAC‐related fibrosis and facilitate tumor progression. Intriguingly, Nicoletta et al. recently elucidated the role of a specific subset of MDMs, denoted as inflammatory, noncytotoxic IL‐1β^+^ TAMs in the progression of PDAC. In particular, they have demonstrated that the exposure of IL‐1β^+^ TAMs to prostaglandin E2 (PGE2) and TNF, induces inflammatory alterations in nearby PDAC cells expressing an IL‐1β^+^ response signature (T1RS), fostering the production of PGE2 and TNF in a positive feedback loop. This perpetuates a functional TME through sustained transcriptional changes associated with tumor progression and correlated with poor patient survival [62].

**Fig. 2:**
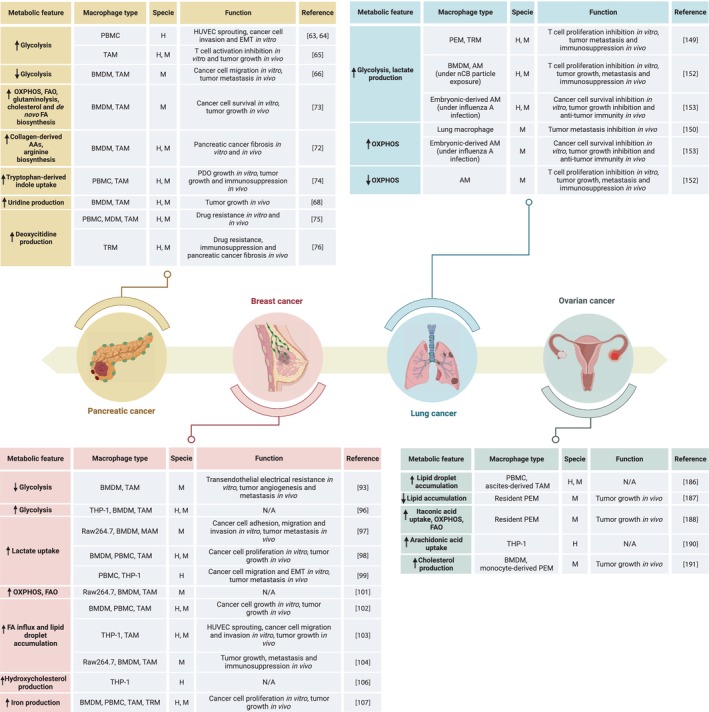
Overview of the metabolic features and tumor‐associated functions of macrophages in different tumor malignancies. Tumor‐associated macrophage metabolism varies upon niche‐specific events and in response to the energy demands of cancer cells and is linked to the distinct functional outcomes observed in pancreatic, breast, lung, and ovarian cancers. AA, amino acid; AM, alveolar macrophage; BMDM, bone marrow‐derived macrophage; EMT, epithelial–mesenchymal transition; FA, fatty acid; FAO, fatty acid oxidation; H, human; HUVEC, human umbilical vein endothelial cell; M, mouse; MDM, monocyte‐derived macrophage; N/A, not applicable; nCB, carbon black ultrafine; OXPHOS, oxidative phosphorylation; PBMC, peripheral blood mononuclear cell; PDO, patient‐derived organoid; PEM, peritoneal macrophage; TAM, tumor‐associated macrophage; TRM, tissue‐resident macrophage.

Several studies have shown that pancreatic cancer cells and their surrounding TME regulate TAM phenotypes via metabolic reprogramming to support tumor development (Fig. 2). Previous investigations demonstrated that PDAC‐educated MDMs display increased glycolytic rates, which are associated with their protumoral and prometastatic phenotype. Interestingly, this pronounced macrophage glucose metabolism was further associated with pancreatic cancer cell growth, extravasation, and invasion [63, 64]. In line with this, specific deletion of glucose transporter 1 (GLUT1) in tumor‐infiltrating macrophages or inhibition of glycolysis by 2‐deoxyglycose (2‐DG) reversed their protumoral profile and decreased PDAC tumor burden [65]. However, another evidence from Zhang et al. has recently shown that PDAC neoplastic cells, through direct cell‐to‐cell interaction of glycoprotein A repetitions predominant (GARP) and integrin αV/β8, induce DNA methylation and downregulation of several glycolysis‐ and OXPHOS‐related genes specifically in proinflammatory macrophages, reprograming them toward an anti‐inflammatory phenotype. Subsequently, this TAM metabolic rewiring activates IL‐10/IL‐10R‐downstream signaling in tumor cells and promotes PDAC cell migration and metastasis formation [66]. This study from Zhang et al. is constrained by the absence of an *in vivo* analysis of glucose metabolism and OXPHOS enzymes as well as polarization markers in TAMs at the tissue level in PDAC. Having these would aid in distinguishing between the anti‐inflammatory macrophages reprogrammed by neoplastic cells and the resident ones. Besides, PDAC glycolytic metabolism was also shown to be influenced by TAMs. Recent data have demonstrated that monocyte‐derived infiltrating macrophages enhance aerobic glycolysis and lactate production in pancreatic cancer by secreting CC motif chemokine ligand 18 (CCL18). This in turn upregulates the expression of the vascular cell adhesion protein 1 (VCAM‐1) in PDAC cells via the membrane‐associated phosphatidylinositol transfer protein 3 (PITPNM3)/NF‐kB axis. Reciprocally, VCAM‐1‐induced lactate production from glycolytic PDAC cells maintains the protumoral phenotype of tumor‐infiltrating macrophages and sustains the positive feedback loop between CCL18‐positive MDMs and PDAC cells [67]. Nonetheless, a recent report from Nwosu et al. [68] suggested that PDAC cancer cells may survive under glucose‐deprived conditions by utilizing TAM‐derived uridine to fuel their central carbon metabolism and drive redox, nucleotide, amino acid and glycosylation metabolite synthesis. This observation further elucidates the intricate dynamics of nutrient accessibility within the TME and underscores the pivotal role of TAM‐derived nucleosides in sustaining cancer cell metabolism, particularly in case of nutrient deprivation.

The aggressiveness of PDAC tumors stems from their markedly desmoplastic stromal nature, characterized by an overgrowth of fibroblasts and immune cells, and by a dense collagen fiber network. Such features form barriers that impede drug delivery and immune cell infiltration, thereby fostering resistance to treatment [69, 70, 71]. Collagen, the major constituent of the extracellular matrix, plays a key role in the functional modulation of TAMs through metabolic rewiring. The results from LaRue et al. [72] ascertained that collagen scavenging‐dependent metabolic reprogramming of macrophages increases arginine biosynthesis, resulting in the upregulation of iNOS and in the production of ROS, which ultimately enhances pancreatic intratumoral fibrosis. Moreover, the Toll‐like receptor 9 agonist, CpG, has been shown to rewire TAM metabolism toward an oxidative phenotype to circumvent the antiphagocytic signals from malignant PDAC cells. The phagocytic and antitumor properties of macrophages induced by CpG were shown to be dependent on FA oxidation and glutaminolysis. Additionally, CpG activation of macrophages redirects exogenous FAs and glucose toward acetyl‐CoA generation, as well as cholesterol and *de novo* lipid biosynthesis [73]. Another study from Hezavah et al. demonstrated that also tryptophan‐derived indoles induce an immunosuppressive phenotype in PDAC‐associated macrophages, through an aryl hydrocarbon receptor (AhR)‐driven mechanism, and suppress T cell maturation. In contrast, AhR abrogation shifts TAM metabolism toward a proinflammatory profile, decreases PDAC tumor growth, and improves the efficacy of immune checkpoint blockade. Importantly, low AhR expression was shown to be beneficial for PDAC patient survival [74].

Mounting evidence suggested paracrine signals between TAMs and PDAC cells within TME modulate their metabolism and promote chemotherapy resistance. Previous reports have demonstrated that macrophages rewire their metabolism to upregulate cytidine deaminase, an enzyme responsible for the breaking down of the nucleoside analog, gemcitabine. Macrophages can also release deoxycytidine (dC), which competes with gemcitabine and, ultimately results in PDAC chemoresistance [75].

Additionally, multiomics analysis of human and mouse pancreatic tissues revealed a decrease in monocyte‐derived APCs, and an increase in proliferating TRMs and inflammatory macrophages after treatment with gemcitabine. Consequently, proliferating TRMs were shown to be significantly associated with poorer PDAC patient outcomes. This subset of macrophages displays chemotherapy tolerance by increasing the production of dC and downregulating dC kinase levels. Importantly, they promote immunosuppression by modulating neighborhood T cells and, together with fibroblasts, lead to PDAC‐associated fibrosis, thereby hindering the effectiveness of chemotherapy [76].

### 2.2 Breast cancer

Macrophages are important players in breast development and homeostasis. Macrophages present in the mammary gland are broadly classified into stromal macrophages (SMs) and ductal macrophages (DMs) and are usually of embryonic origin. Stromal macrophages are characterized by CD11c^lo^, CD11b^+^, Ly6C^+^, MHCII^hi/lo^ expression, whereas the ductal macrophages are CD11c^+^, CD11b^lo^, Ly6C^−^, Cx3cr1^hi^ [77, 78]. Throughout an individual's lifespan, SMs are gradually replaced by MDMs, whereas the majority of DMs are swiftly replaced at puberty and transformed as long‐lived cells [77].

In breast cancer, TAMs constitute more than 50% of the total immune cell infiltrate and support tumor progression by promoting angiogenesis, immune evasion, and metastasis [18, 79, 80]. Early breast tumors are enriched in locally proliferating CD11b^−^ DMs however, advanced tumor lesions are mainly populated by CD11b^+^ DMs, which are associated with a poor prognosis [81]. Moreover, during breast cancer evolution, inflammatory monocytes are recruited to the tumor niche, where they differentiate into TAMs. These TAMs express MMP‐9 and release cytokines, such as TGF‐β and IL‐10 that promote angiogenesis and an immunosuppressive TME. Besides, cancer cells and other stromal cells orchestrate signals to modulate macrophage phenotype and function which collectively drives breast carcinogenesis [82].

Breast TAMs constitute a mixed population of TRMs and MDMs; however, it remains unclear which population contributes to the majority of the protumoral TAM pool [83, 84]. Reed et al. [85] suggested a predominant role for TRMs, as they observed that even after the depletion of MDMs, an aggressive breast cancer phenotype persisted. Furthermore, Hirano et al. [86] reported that mammary TRMs constitute the major source of TAMs in triple‐negative breast cancer (TNBC) and that their depletion significantly reduces tumor growth, recurrence, and metastasis. In line with this, some studies have demonstrated that during the earlier stages of breast cancer progression, TRMs display a proinflammatory profile; however, as the tumor progresses, they switch to an anti‐inflammatory phenotype that fosters breast cancer dissemination [87, 88, 89, 90]. Nonetheless, a rather contrasting study from Bonapace et al. [91] suggested a leading role of MDMs in breast tumor progression and aggressiveness, by demonstrating that increased expression of CCL2, a monocyte chemoattractant, is correlated with poor patient survival. Moreover, in an MMTV‐PyMT model, it was shown the recruitment and infiltration of macrophages at the primary tumor and at the lung metastatic site is dependent on the CCL2/CCR2 axis [24]. An interesting study from Franklin et al. [92] also showed that TAMs differentiate from CCR2^+^ inflammatory monocytes and that depletion of TAMs and not TRMs suppresses breast tumor growth. These findings collectively suggest that the function of TRMs and MDMs in breast cancer progression is dynamic and may be influenced by the stage of cancer development. TRMs may have a predominant role early on, while MDMs might become more crucial in later stages. These data further underscore the complexity of TAMs' contributions to breast cancer and emphasize the need for further research to understand the precise mechanisms and therapeutic implications associated with these macrophage populations in breast cancer.

The protumoral phenotype of TAMs usually observed in breast cancer models is linked with alterations in their metabolism (Fig. 2). Growing evidence has unveiled that breast cancer cells manipulate various metabolic pathways in TAMs to their advantage, as exemplified by glycolysis [34]. TAMs supporting breast cancer progression are known to display reduced glycolytic rates however, other studies suggested the opposite can also be observed [93, 94, 95]. According to the findings of Wenes et al. [93], regulated in development and DNA damage response 1 (REDD1), an mTOR inhibitor, shifts the metabolism of hypoxic TAMs toward reduced glycolysis, which promotes neoangiogenesis and metastasis in a murine breast cancer model. However, the significance of these alterations in glucose metabolism has not been elucidated in humans yet. However, Liu et al. [96] reported that bone marrow‐derived macrophages (BMDMs) incubated with tumor extracts from breast cancer patients show elevated levels of Hexokinase‐2 and other glycolytic enzymes, indicating that breast cancer cells may increase rather than decrease glycolysis in macrophages and this reprogramming was associated with enhanced tumor progression. Therefore, despite increased competition for local glucose availability, it is likely that glycolysis in TAMs may still contribute to tumor growth. Breast cancer cell‐derived lactate favors the anti‐inflammatory polarization of TAMs by activating G‐protein‐coupled receptor 132 (GPR132). Increased levels of GPR132 promote the infiltration of protumoral MDMs into breast tumors, while the pharmacological inhibition and genetic deletion of GPR132 reduce tumor growth and metastasis [97, 98]. Specifically, Lin et al. [99] showed the protumoral phenotype of TAMs induced by cancer cell‐derived lactic acid increases the secretion of CCL5, which consequently stimulates aerobic glycolysis in breast cancer cells and promotes cell migration and invasiveness. However, anti‐inflammatory BMDMs support breast cancer cell glycolysis by secreting TGF‐β, which downregulates succinate dehydrogenase and enhances HIF1‐α‐stabilization in breast cancer cells. Additionally, protumoral macrophages promote PD‐L1 expression and HIF1‐α‐induced vascularization in 4T1 breast tumors, resulting in increased angiogenesis and immunosuppression [100]. These orchestrated effects, including PD‐L1 expression, HIF1‐α‐induced vascularization, angiogenesis, and immunosuppression by protumoral macrophages in 4T1 breast tumors, further highlight the intricate metabolic and immunomodulatory dynamics within the TME. Future research in this realm holds promise for targeted interventions aimed at disrupting these interactions for therapeutic benefit in breast cancer.

Besides alterations in glycolytic metabolism, protumoral TAMs have been demonstrated to rewire their lipid metabolism to support breast cancer progression. Dominique et al. have identified a novel role for Hedgehog signaling in the metabolic regulation of anti‐inflammatory TAMs, by supporting FA oxidation. In fact, the pharmacological inhibition of Hedgehog signaling was shown to shift TAM metabolism from FA oxidation to glycolysis and consequently, promote macrophage repolarization from an anti‐inflammatory to a proinflammatory phenotype [101]. Moreover, Hao et al. found that the protumoral phenotype of macrophages can also be induced by the expression of certain fatty acid‐binding proteins (FABP). During the early stages of human breast cancer, recruited inflammatory macrophages express FABP5, a regulator of lipid droplet accumulation and immunostimulatory cytokine secretion. However, during the late stages of breast cancer progression, macrophages exhibit high FABP4 expression, which facilitates IL‐6/STAT3 signaling and promotes the anti‐inflammatory phenotype of macrophages [102]. In addition, another study from Niu et al. [103] demonstrated that the accumulation of lipid droplets, via Caspase‐1/tPPARγ/medium‐chain acyl‐CoA dehydrogenase signaling axis, in TAMs isolated from mouse breast cancer tissues and human MDMs cocultured with MCF‐7 breast cancer cells, enhances lactate secretion and reprograms TAMs toward an anti‐tumoral phenotype. Xiang et al. also showed that lipid accumulation in TAMs, by downregulation of monoacylglycerol lipase, which hydrolyses triglycerides into free FAs, promotes anti‐inflammatory activation of TAMs and tumor progression [104].

Breast cancer cells rely on TAM cholesterol metabolism to support their proliferation [105]. In fact, Shi et al. demonstrated that anti‐inflammatory TAM‐produced 27‐hydroxycholesterol increases the proliferation of ER^+^ breast cancer cells and supports their pathology [106]. In addition, Mertens et al. [107] revealed that breast cancer cells can also take advantage of TRM‐derived iron for their proliferation and identified lipocalin‐2 (LCN‐2) as a critical iron transporter in this context. In line with this study, Ören et al. [108] reported that macrophage release of LCN‐2 promotes breast cancer metastasis.

A compelling avenue for future exploration lies in deciphering the intricate interplay between lipid metabolism and the phenotypic plasticity of TAMs in breast cancer. Further investigation into the dynamics of specific FABPs, such as FABP5 and FABP4, and their differential expression in distinct stages of breast cancer could unveil nuanced regulatory mechanisms that impact TAM behavior. The implications of lipid droplet accumulation in TAMs, as highlighted by studies involving Caspase‐1/tPPARγ/medium‐chain acyl‐CoA dehydrogenase signaling and monoacylglycerol lipase downregulation, present promising directions for understanding the potential role of lipid metabolism in reprogramming TAMs towards an anti‐tumoral phenotype. As we delve deeper into these interactions, the identification of key signaling axes and regulatory nodes may open avenues for targeted therapeutic interventions in breast cancer progression.

### 2.3 Lung cancer

Murine and human lungs comprise three major populations of resident macrophages: alveolar macrophages (AMs), interstitial macrophages (IntMs), and nerve‐associated macrophages (NAMs). AMs are localized in the alveoli, IntMs exist in the bronchial interstitium close to the blood vessels and NAMs are found in the alveolar interstitium and in the peribronchial next to the nerves [109, 110, 111, 112, 113]. All of them are derived from yolk sac macrophages and fetal liver monocytes; however, AMs are maintained independently of circulating monocytes, whereas the IntMs and NAMs are gradually replaced by circulating monocytes after birth [114, 115]. AMs engage in phagocytosis of inhaled particles and conduct immune surveillance, but their primary role is the clearance of pulmonary surfactant [114, 116, 117, 118, 119]. IntMs maintain lung homeostasis by preventing immune‐driven allergic responses [120, 121, 122, 123]. In physiological conditions, IntMs are significantly fewer in number than AMs and the distinction of these two macrophage subtypes becomes less evident during homeostasis disruption. Although both AMs and IntMs express the conventional pro‐/anti‐inflammatory‐related markers, they do exhibit distinct metabolic profiles [111, 124, 125, 126, 127]. AMs display increased OXPHOS and lipid catabolism, while IntMs are more glycolytic in nature and exhibit decreased mitochondrial respiration, FA, and cholesterol metabolism [128, 129, 130, 131, 132].

In lung cancer, macrophages constitute the major leukocyte infiltrate (30–50% of stromal cells) and play a pivotal role in the malignant progression of most lung cancer subtypes, with lung adenocarcinoma (LUAD) harboring the highest infiltrate of macrophages [16, 133, 134]. As already described for previously mentioned cancers, at early stages of lung cancer, TAMs mainly exhibit a proinflammatory/antitumoral profile however, as the tumor progresses, they switch into an anti‐inflammatory/protumoral phenotype [135, 136]. Nonetheless, He et al. reported that in early LUAD, macrophages do not exhibit an exclusive pro‐ or anti‐inflammatory signature, rather they express similar levels of pro‐/anti‐inflammatory‐associated genes [137]. Single‐cell RNA‐seq analyses in tumor and adjacent nontumor tissues of nonsmall‐cell lung cancer (NSCLC) patients have reported distinct phenotypic and functional characteristics of macrophage subpopulations [138, 139, 140]. TRMs have been reported to aid in conditioning the protumorigenic niche at early stages of NSCLC [140]. In line with this, a study from Casanova‐Acebes et al. revealed the depletion of TRMs, prior to tumor inoculation in a murine KRAS‐mutated lung cancer model, enhanced CD8^+^ T‐cell infiltration and reduced tumor burden. Nonetheless, when TRMs were depleted after tumor inoculation, no effects were found [140]. Other studies also showed that, during early lung tumor progression, TRMs drive tissue remodeling programs and tumor cell invasion. As the tumor progresses, TRMs redistribute to the tumor periphery and MDMs become the dominating population of TAMs within the tumor milieu [137, 138, 139]. Moreover, in metastatic lesions of LUAD, there is a significant abundance of MDMs, which directly correlates with both tumor stage and metastasis. Interestingly, these MDMs were shown to preferentially exhibit a protumoral phenotype [139, 141]. This dynamic interplay between TRMs and MDMs highlights the importance of understanding the temporal and spatial aspects of macrophage involvement in lung cancer, which may have implications for understanding disease mechanisms and develop targeted therapeutic strategies.

Macrophage metabolism has been shown to play a critical role in shaping lung cancer cell metabolism as well as immune responses (Fig. 2). In patients with NSCLC, TAMs were found to be more oxidative and to promote tumor cell glycolysis by secreting TNF‐α. Moreover, the activation of AMP‐activated protein kinase (AMPK) and peroxisome proliferator‐activated receptor‐γ coactivator (PGC‐1α) in TAMs exacerbates tumor hypoxia and leads to a decreased PD‐L1 expression and T‐cell infiltration into lung tumors [142, 143]. Interestingly, the oxidative metabolism of tumor‐promoting TAMs was shown to be dependent of fatty acid oxidation [144]. This metabolic preference actively contributes to the creation of an immunosuppressive TME, ultimately facilitating the immune evasion of tumor cells and resistance to therapies [145]. In the context of malignant pleural effusion and lung cancer, Zhang et al. [146] showcased that C1q^+^ macrophages exert protumoral effects by enhancing fatty acid metabolism, leading to the promotion of immunosuppression through the upregulation of FABP5 and activation of PPARγ.

Primary lung tumor‐derived soluble factors, such as cytokines and tumor‐derived exosomes (TDEs), were shown to reprogram TAM metabolism and promote a protumoral and immunosuppressive phenotype that aids in premetastatic niche formation [147, 148]. A study by Samantha et al. has reported that in a murine Lewis lung carcinoma model, TDEs support metastasis formation by reprogramming interstitial TRM metabolism. TDEs increase NF‐kB‐mediated glycolysis and lactate production, regulate iNOS‐mediated inhibition of oxidative metabolism, and drive macrophages toward an immunosuppressive phenotype [149]. A rather contrasting study from Kersten et al. [150] recently reported the ingestion of tumor‐derived microparticles, including exosomes and extracellular vesicles, induces a metabolic switch in MDMs towards OXPHOS via mTORC1 signaling in the early metastatic lung, supporting an antimetastatic function in the premetastatic lung. Nonetheless, as the metastasis advances, the initially observed antitumoral characteristics gradually diminish. This transition in macrophage phenotype can be attributed to the increasing metabolic requirements of metastatic lungs, potentially causing spatiotemporal alterations in the nutrient composition of TME that drive the reprogramming of macrophage function and phenotype [142]. Moreover, the discrepancy with the former study may be attributed to the timing of the investigation, as the latter study exclusively considered the initial phases of the interaction between macrophages and cancer cells in the lung.

Among various regulatory entities, long noncoding RNAs (lncRNAs) are one of the potent modulators orchestrating processes that potentially impact tumor progression and immune response. LncRNAs are overexpressed in various cancers, and exhibit high cell and tissue specificity, with certain variants specifically expressed in macrophages. Karger et al. reported that the overexpression of lncRNA ADPGK‐AS1 in M2‐like human macrophages and TAMs from human lung cancer tissues alters their metabolic phenotype. ADPGK‐AS1‐overexpressed macrophages manifest an M2‐like phenotype and exhibit increased levels of TCA cycle metabolites but reduced lactate content, while macrophage‐specific knockdown of ADPGK‐AS1 induces phenotypic and metabolic reprogramming toward an M1‐like phenotype, ultimately inhibiting lung tumor growth [151].

Besides tumor‐derived factors, external elements, such as carcinogens and viral infections, were shown to indirectly contribute to steering macrophage metabolism toward a protumoral phenotype. An interesting study from Chang et al. reported that chronic exposure to carbon black ultrafine (nCB) particles, often present in the lungs of habitual smokers, metabolically rewires lung macrophages to promote an immunosuppressive TME and accelerate NSCLC progression. Intriguingly, nCB‐exposed macrophages showed damaged mitochondria with impaired OXPHOS, and increased HIF1‐α signaling, glycolysis, and lactate production. This resulted in an increased abundance of immunosuppressive T cells and in the development of lung tumors and distant metastasis [152]. Furthermore, Wang et al. have also reported that influenza A‐infected TRMs develop phagocytic and tumor cell‐cytotoxic functions, providing lungs with a long‐lasting antitumor immunity. This trained immunity of macrophages is sustained by prolonged metabolic rewiring which involves alterations in glycolytic, phosphoinositide 3‐kinase (PI3K)‐Akt, HIF1‐α, and mTOR pathways as well as increased oxygen consumption and extracellular acidification rates [153].

Further investigations are warranted to elucidate the mechanisms governing the phenotypic and metabolic transitions of macrophages throughout different stages of lung cancer progression. The contrasting findings regarding the impact of TDEs on macrophage metabolism prompt speculation about the contextual factors and temporal dynamics influencing their responses. Additionally, the intriguing connections between external elements, such as chronic exposure to nCB particles and viral infections, and their ability to define macrophage metabolism open avenues for future exploration.

### 2.4 Ovarian cancer

Macrophages are found in abundance in mammalian ovaries, where they perform specialized functions in maintaining tissue homeostasis. Similar to other tissues, ovarian macrophages exhibit a significant level of developmental diversity and have multiple origins. During embryonic development, macrophages originating from both the yolk sac and fetal liver colonize the ovary. However, after birth, bone marrow‐derived monocytes also contribute to the ovarian macrophage pool [154, 155].

Macrophages constitute the primary immune cell population, accounting for around 39% of immune cells within the ovarian TME, where they exhibit different phenotypes and functions depending on their origin or polarization status [156, 157, 158, 159, 160]. TAMs play a pivotal role across the various stages of ovarian cancer development by promoting cancer cell growth, immune evasion and metastasis [161, 162, 163, 164]. While certain reports have indicated the co‐expression of pro‐/anti‐inflammatory markers in ovarian TAMs, others have shown that they predominantly display a protumorigenic phenotype [157, 165, 166, 167]. Nevertheless, infiltration of anti‐inflammatory TAMs in ovarian tumors has been associated with tumor aggressiveness and resistance to therapy [161, 162, 163, 164]. The transition of TAMs towards a pro‐tumoral phenotype was shown to be mediated by cancer cells and several constituents of the TME, including interleukins, growth factors, and metabolites [156, 168]. For instance, ovarian cancer cell‐derived mucins and microRNAs were demonstrated to activate TAMs towards a protumoral state that contributes to an immunosuppressive TME [169, 170, 171]. In turn, TAMs activate prosurvival signaling pathways in ovarian cancer cells by releasing cytokines, such as IL‐6 and IL‐10 [172].

As with the other types of cancer mentioned above, ovarian tumor macrophages consist of a population of resident and monocyte‐derived macrophages. MDMs have been shown to promote ovarian cancer cell survival by activating the STAT3 signaling pathway, whereas TRMs have been associated with ovarian cancer cell proliferation and invasion of the metastatic niches at the peritoneal cavity and omentum [172, 173, 174, 175]. A comparative analysis of MDMs, resident peritoneal macrophages, and human ovarian ascetic TAMs revealed that TAMs are phenotypically and functionally more similar to resident peritoneal macrophages than MDMs. These common traits included similarities in their transcriptomes, immune activation state and function, and expression of tumor‐promoting mediators [176]. Xia et al. demonstrated that the peritoneal fluid in an ID8 ovarian cancer model is populated by two macrophage subpopulations with differential expression of Tim‐4. Tim‐4^+^ TAMs originate during embryonic development, are maintained locally and are the predominant population of F4/80^hi^MHC‐II^lo^ subset, whereas Tim‐4^−^ TAMs are replenished by circulating monocytes and represent the majority of F4/80^lo^MHC‐II^hi^ subset. Nonetheless, while Tim‐4^−^ TAMs did not show a significant role in ID8 tumor development, Tim‐4^+^ TAMs actively supported tumor progression by exhibiting elevated expression of arginase 1, increased mitophagy activity, and reduced mTORC1 activity [177]. In addition to resident peritoneal macrophages, another type of TRMs that have been implicated in ovarian cancer cell dissemination are omental macrophages [178]. Yin et al. [175] revealed that TRM‐induced implantation of ovarian ascetic spheroids onto the omentum is mediated by the secretion of protein‐remodeling factors, such as MMP‐9, vascular endothelial growth factor (VEGF)‐C and TGF‐β. Moreover, a study by Anders et al. reported that a specific subset of resident omental macrophages, identified as CD163^+^Tim4^+^, play a critical role in the metastasis of ovarian cancer. In fact, the genetic and pharmacological depletion of CD163^+^Tim4^+^ macrophages and not MDMs regressed the metastatic spread of ovarian cancer [179]. Interestingly, the production of colony‐stimulating factor‐1 (CSF‐1), hepatocyte growth factor (HGF), and epidermal growth factor (EGF) by ovarian cancer cells, endothelial cells, and TAMs, respectively, and the formation of a paracrine loop was shown to be crucial for metastatic progression [175, 178, 180]. In particular, TAM‐derived EGF has been shown to sustain the formation of ascetic spheroids during metastasis by facilitating their interaction with ovarian cancer cells, which is mediated by αMβ2 integrin and intercellular adhesion molecule 1 (ICAM‐1) [175].

Numerous studies have demonstrated that ovarian cancer cells and their surrounding TME orchestrate the metabolic reprogramming of TAMs to reinforce ovarian cancer initiation and growth (Fig. 2). Within the TME, ovarian cancer cells and TAMs engage in a metabolic competition for limited nutrients, such as glucose and glutamine. The high demands of ovarian cancer cells for glucose results in an increased lactate production that promotes acidification of TME and consequently, the metabolic reprogramming of TAMs [155, 181, 182]. Bi et al. [183] have documented a positive correlation between the ovarian cancer cell expression of the glycolytic genes decorin 1 (DCN1) and fructose bisphosphatase 1 (FBP1) in ovarian cancer cells and macrophage infiltration as well as the TME immune score. Similar to glucose, cancer cells exhibit a dependency on glutamine for their nutritional needs, utilizing it to fuel their proliferation [184]. This glutamine addiction by ovarian cancer cells fosters the production and release of N‐acetyl aspartate which, in turn, promotes the anti‐inflammatory phenotype of macrophages [185].

Besides, ovarian TAMs also exhibit an altered lipid metabolism, encompassing increased FA production, absorption, and storage. This metabolic rewiring has been associated with functional reprogramming by ovarian cancer cells; however, the specific mechanisms involved remain largely unclear [37]. Lipidomic analysis of human ovarian cancer samples by Schumann et al. [186] suggested that ovarian cancer ascites induce a protumoral polarization of TAMs by promoting lipid droplet accumulation through the PPARβ/δ activation with polyunsaturated fatty acids (PUFAs), especially linoleic acid. Furthermore, Casanova‐Acebes et al. also suggested the lipid metabolism of resident peritoneal macrophages can be regulated by retinoid X receptors (RXRs). Deletion of RXRα&β led to excessive lipid accumulation in Tim4^+^ resident peritoneal macrophages, which reduced their survival and accumulation in early ovarian tumors and slowed primary ovarian tumor growth [187]. Moreover, Weiss et al. demonstrated that, in ID8 intraperitoneal tumors, F4/80^+^ TRMs express high levels of immune‐responsive gene 1 (IRG1) and itaconic acid and display heightened oxidative phosphorylation. Interestingly, IRG1 deletion in resident peritoneal macrophages leads to decreased OXPHOS and subsequently, reduced ROS production. This, in turn, attenuates ROS‐mediated MAP kinase activation in tumor cells and suppresses the ovarian tumor growth [188]. However, ovarian cancer cell lipid metabolism was also shown to be influenced by TAMs. Yu et al. [189] showed that macrophage‐derived IL‐17A modulates adipocyte FA uptake and metabolism and stimulates ovarian cancer cell growth and metastasis. Wen et al. [190] have reported that increased hypoxic ovarian cancer cell production of 5‐lipoxygenase‐related metabolites, such as arachidonic acid, promote migration and invasion of macrophages *in vitro*, by upregulating the p38/MMP‐7 pathway, and is positively correlated with density of TAMs in hypoxic areas. Furthermore, ovarian cancer‐associated TAMs also exhibit altered cholesterol metabolism. Pieter et al. observed that monocyte‐derived TAMs display upregulated cholesterol metabolism. Ovarian cancer cells prompt the release of cholesterol from TAMs, boost IL‐4 signaling, and suppress interferon (IFN)‐γ‐induced gene expression. These IL‐4 signaling and cholesterol efflux pathways in TAMs significantly contributed to ovarian cancer progression [191].

Overall, this intricate metabolic interplay between ovarian cancer cells and TAMs within the TME is a pivotal determinant of ovarian cancer initiation and progression. Unraveling the precise mechanisms governing these reciprocal metabolic interactions may provide novel therapeutic targets for disrupting this metabolic symbiosis that fuels ovarian cancer progression, paving the way for innovative strategies in metabolic targeted therapies for ovarian cancer.

## 3 Conclusions and perspectives

Tumor‐associated macrophage heterogeneity is a result of their adaptation to their associated microenvironment as well as a reflection of their cellular ontogeny. In fact, several studies point out how TAM function and metabolism varies upon niche‐specific signaling and metabolic events across different types of cancers and how their phenotypic characteristics promote tumor growth and metastasis. Generally, macrophages are broadly classified into two functionally contrasting subtypes: proinflammatory/antitumoral macrophages, which exhibit a more glycolytic phenotype, and anti‐inflammatory/protumoral macrophages that use oxidative phosphorylation to generate energy. This is, however, an oversimplification since TAMs cannot be described entirely by this nomenclature as their overall functional and metabolic profile has been shown to be very dynamic and complex to define. While we have tried to elucidate the metabolic profile of TAM subsets in pancreatic, lung, breast, and ovarian cancers, there are some studies which present a contradictory view. That can be attributed to the complex and dynamic nature of the TME as well as the specific experimental conditions and contexts in which the studies were conducted. Understanding the nature of context‐dependent interactions between cancer cells and macrophages is crucial for the discovery of the underlying mechanisms and for the development of targeted therapies. Originally, TAMs were thought to be derived exclusively from circulating monocyte precursors; however, recent studies have clearly implicated the involvement of resident macrophages as well. Monocyte‐derived macrophages are believed to be more pro‐tumoral in nature and display oxidative phosphorylation. Although it is well known that resident macrophage phenotype is correlated with the host tissue function, their function and metabolism are still controversial with some reports suggesting an antitumoral phenotype, whereas others define them as protumoral. Furthermore, the absence of distinct markers for distinguishing monocyte‐derived from resident macrophage TAMs adds complexity to discerning their phenotype and function. A clear understanding of the metabolic regulation of these different TAM subsets is also limited since it is challenging to quantify the temporal and spatial distribution of metabolites in tissues without disturbing the tissue architecture. The conventional *in vitro* systems, meanwhile, do not provide the spatial structure that could mimic the heterogeneity of metabolic environments. Although there is still a long way to go towards clarifying the metabolic fluxes and the metabolic interconnections between macrophage subsets and cancer/stromal cells within the TME, targeting the metabolism of TAMs has gained increased attention in the last years. Increasing evidence suggests the metabolic re‐education of TAMs can repolarize them toward an antitumoral phenotype. Nevertheless, the efficacy of this approach is limited due to acquired resistance or compensatory mechanisms. Moreover, the shared metabolic profiles of different TME cells may lead to undesired effects. Therefore, a complementary combination of more cell‐specific metabolic interventions and immunotherapeutic strategies can be harnessed to mediate anticancer effects.

## Conflict of interest

The authors declare no conflict of interest.

## Author contributions

UA, AP‐N, and MM contributed to conceptualization; UA and AP‐N contributed to writing; UA, AP‐N, MD, and MM contributed to critical insights and edits to the text. All authors read and approved the manuscript.
